# Tumor purity as a prognosis and immunotherapy relevant feature in gastric cancer

**DOI:** 10.1002/cam4.3505

**Published:** 2020-10-08

**Authors:** Zhe Gong, Jieyun Zhang, Weijian Guo

**Affiliations:** ^1^ Department of Medical Oncology Fudan University Shanghai Cancer Center Shanghai P.R. China; ^2^ Department of Oncology Shanghai Medical College Fudan University Shanghai P.R. China

**Keywords:** gastric cancer, prognosis, stromal and immune scores, tumor microenvironment, tumor purity

## Abstract

Tumor microenvironment (TME) has been illustrated their clinic pathological significance in predicting outcomes and therapeutic efficacy by more and more studies. Tumor purity, which reflects the features of TME, is defined as the proportion of cancer cell in the tumor tissue. However, the current staging and prognostic prediction system in gastric cancer (GC) paid little attention to TME. Therefore, we carried out the study to explore the role of tumor purity in GC. We retrospectively collected the clinical and transcriptomic data from four public data sets (n = 1340), GSE15459, GSE26253, GSE62254, and The Cancer Genome Atlas (TCGA). About 34 GC patients from Fudan University Shanghai Cancer Center (FUSCC) were assigned as an independent validation group. Tumor purity was measured by a computational method. Low tumor purity was associated with unfavorable prognosis, upregulated EMT and stemness pathways, more infiltrating of Tregs, M1 and M2 macrophages and a higher expression level of various immune checkpoints and chemokines recruiting immune suppressive cells. Our study indicates low tumor purity in GC was associated with unfavorable prognosis and immune‐evasion phenotype. Further investigations toward tumor purity in GC may contribute to prognosis prediction and the decision of therapy strategies.

## Graphical abstract

Tumor purity plays an important role in prediction of prognosis and genomic conditions in gastric cancer. Low purity in GC was associated with enhanced immune evasion and poor prognosis, which indicated that low purity gastric cancer patients may benefit more from immunotherapy.

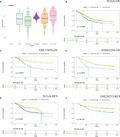

## 1 BACKGROUND

Nowadays, gastric cancer (GC) is a common and one of the most leading causes of cancer‐related mortality worldwide.^1^ As the most widely used staging method, TNM staging system is based on depth of infiltration, local lymph node metastases and distal metastases. However, some patients have the same TNM stage but the distinct clinical outcomes.^2^ Therefore, it is urgent to identify the underlying key factors for predicting prognosis in GC.

Tumor purity is defined as the proportion of cancer cells in the tumor tissue, which reflects the characteristics of TME. Yoshihara et al.^3^ have developed ESTIMATE algorithm for assessment of the presence of stromal cells and the infiltration of immune cells in tumor samples using gene expression data and further calculating tumor purity. ESTIMATE algorithm was proved as a robust algorithm for tumor purity prediction. Previous studies^4^, ^5^ revealed that low tumor purity was associated with unfavorable prognosis in colon cancer and glioma. However, few studies focused on tumor purity in GC. Thus, we carried out this study to explore the association between tumor purity and clinical prognosis in GC and its mechanisms. Further, we used tumor purity to predict clinical benefits in GC patients treated with immunotherapy. We hope the analyses of tumor purity in GC could provide a novel insight in prognosis predicating and treatment strategies.

## 2 METHODS

### 2.1 Study samples

About 407, 200, 432, 300, and 34 GC patients form TCGA data set, GSE15459, GSE26253, GSE62254, and FUSCC, respectively, and 37 GC cell lines from Cancer Cell Line Encyclopedia (CCLE, https://portals.broadinstitute.org/ccle) were enrolled in our study. The detailed criteria and follow up procedures are described in the Additional File 1: Document S1. Patient characteristics of the two cohorts were described in Table 1. The detailed information is described in the Additional File 1: Document S1.

**Table 1:** Patient characteristics of TCGA and FUSCC cohorts

Variable	TCGA cohort				FUSCC cohort			
	Low purity (N = 203)		High purity (N = 204)		Low purity (N = 17)		High purity (N = 17)	
Median purity	64.04%		86.51%		55.63%		81.90%	
	N	%	N	%	N	%	N	%
Sex								
Male	125	61.58	136	66.67	16	94.12	14	82.35
Female	78	38.42	68	33.33	1	5.88	3	17.65
Age								
<56	38	18.72	30	14.71	5	29.41	5	29.41
≥56	163	80.30	173	84.80	12	70.59	12	70.59
Unknown	2	0.99	1	0.49	0	0.00	0	0.00
TNMstage								
1	17	8.37	36	17.65	9	52.94	11	64.71
2	65	32.02	57	27.94	8	47.06	6	35.29
3	85	41.87	81	39.71	0	0.00	0	0.00
4	20	9.85	21	10.29	0	0.00	0	0.00
Unknown	16	7.88	9	4.41	0	0.00	0	0.00
pT								
1	2	0.99	18	8.82	14	82.35	14	82.35
2	42	20.59	44	21.57	0	0.00	0	0.00
3	88	43.35	91	44.61	0	0.00	1	5.88
4	63	31.03	50	24.51	3	17.65	2	11.76
Unknown	8	3.94	1	0.49	0	0.00	0	0.00
pN								
0	56	27.59	63	30.88	7	41.18	10	58.82
1	58	27.59	52	25.49	5	29.41	4	23.53
2	39	19.21	39	19.12	2	11.76	0	0.00
3	40	19.70	41	20.10	3	17.65	3	17.65
Unknown	10	4.93	9	4.41	0	0.00	0	0.00
pM								
0	180	88.67	182	89.22	17	100.00	17	100.00
1	15	7.39	12	5.88	0	0.00	0	0.00
Unknown	8	3.94	10	4.90	0	0.00	0	0.00
Histology type								
Other adenocarcinoma	178	87.68	194	95.10	15	88.24	16	94.12
Mucinous adenocarcinoma/Signet ring cell carcinoma	23	11.33	9	4.41	2	11.76	1	5.88
Unknown	2	0.99	1	0.49	0	0.00	0	0.00

### 2.2 RNA sequencing

To get the RNA‐seq data in FUSCC cohort, we treated the total RNA samples of GC tissue with Ribo‐off rRNA Depletion Kit (Vazyme) in order to construct the RNA‐seq libraries. The detailed information is described in the Additional File 1: Document S1.

### 2.3 Bioinformatic analysis

We used ESTIMATE R package to infer tumor purity in gastric tumor tissue. GISTIC 2.0 was utilized to analyze the copy number alterations (CNA) events. DAVID’s Functional Annotation Clustering module was used to classify gene list into functional‐related gene groups. CIBERSORT algorithm was utilized to estimate the absolute score and relative proportion of 22 immune cells for each sample in TCGA cohort. The results of cell type enrichment analysis for TCGA data using xCell^6^ were downloaded from https://xcell.ucsf.edu/. Gene set enrichment analysis (GSEA) was performed by the GSEA software v.3.0. The detailed information is described in the Additional File 1: Document S1.

### 2.4 Statistical analysis

All of our analyses were conducted using R software version 3.5.2 (https://www.r‐project.org/) and SPSS 20.0 (SPSS Inc., Chicago, IL, USA). Kaplan‐Meier analyses was used to evaluate the relationship between different purity groups and overall survival (OS). Univariate and multivariate Cox regression analyses were performed to identify independent prognostic factors. Student's *t* tests was used to compare variables between groups. Correlations between categorical variables were evaluated by chi‐square analyses. *p* value <0.05 was admitted statistically significant.

See the Additional File 1: Document S1 for other descriptions of the materials and methods used in this study.

## 3 RESULTS

### 3.1 Associations between tumor purity and clinical characteristics and patients’ prognosis

**Figure 1:**
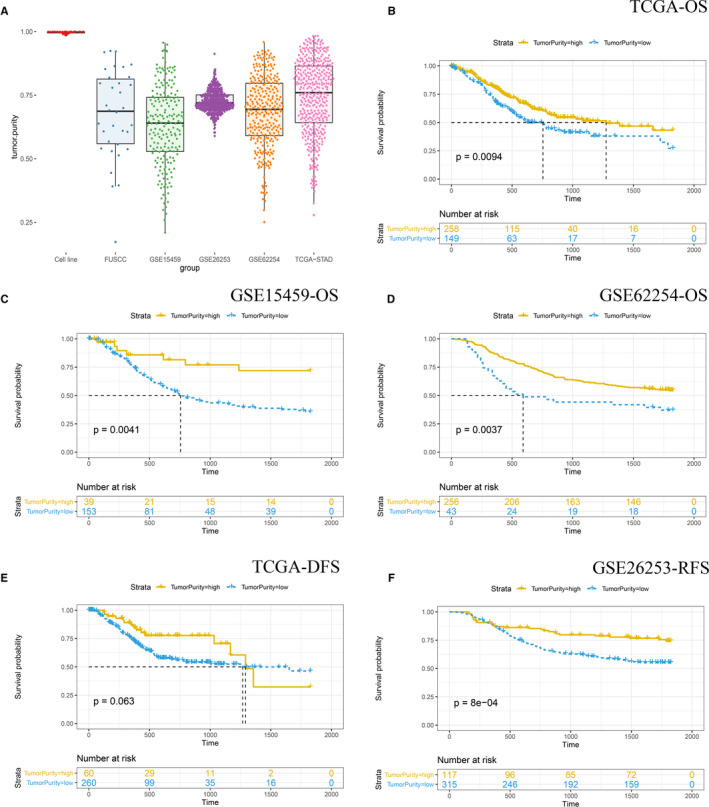
(A) The distribution of tumor purity in 37 cell lines, GSE15459, GSE26253, GSE62254, FUSCC cohort, and TCGA cohort. Kaplan‐Meier analysis of overall survival showed low purity gastric cancer (separated by cutoff tumor purity calculated by ROC analyses) that conferred worse prognosis in TCGA (B), GSE15459 (C), and GSE62254 (D) cohort. Kaplan‐Meier analysis of DFS and RFS showed low purity gastric cancer (separated by cutoff tumor purity calculated by ROC analyses) that conferred worse prognosis in TCGA (E), and GSE26253 (F) cohort, respectively

We performed the ESTIMATE algorithm to calculate stromal and immune scores, which form the basis for the ESTIMATE score to infer tumor purity. For the 37 GC cell lines, the median tumor purity was 99.84% (range: 98.58%‐100.00%). Among patients from TCGA, GSE15459, GSE26253, GSE62254, and FUSCC the median tumor purity was 76.02% (range: 27.84%‐98.34%), 64.03% (range: 20.96%‐95.67%), 71.98% (range: 58.97%‐91.27%), 69.42% (range: 25.09%‐95.93%), and 68.69% (range: 17.26%‐92.43%), respectively (Figure 1A). Chi‐square analyses was performed in TCGA cohort to explore the association between tumor purity and clinical characteristics and the results showed that low purity was associated with more mucinous adenocarcinoma and signet ring cell carcinoma (*p* < 0.05). TCGA cohorts, GSE15459 and GSE62254 were divided into high‐purity and low‐purity groups with the cut‐off 69.06%, 75.71%, and 52.45% calculated by receiver operating characteristic (ROC) analyses, respectively. As shown in Figure 1B‐D, the Kaplan‐Meier survival curves showed high purity conferred prognostic benefit (all *p* < 0.05). Furthermore, Figure 1E,F showed the Kaplan‐Meier survival curves of disease‐free survival (DFS) and recurrence‐free survival (RFS) in TCGA cohort and GSE26253, respectively (patients were divided into high‐purity and low‐purity groups with the cut‐off 88.46% and 74.96% calculated by ROC analyses, respectively), and the results showed low tumor purity was associated with more recurrence and metastasis, although the Kaplan‐Meier survival analyses of DFS in TCGA cohort did not reveal statistically significant (*p* = 0.063). Moreover, we performed univariate Cox regression (Table 2) and found tumor purity, age, TNM stage were associated with OS (all *p* < 0.05). Thus, a multivariate Cox analysis was performed, and tumor purity was identified as an independent prognostic indicator regardless of age and TNM stage (*p* = 0.007, HR =1.587) (Table 2). For the FUSCC cohort, 31 patients’ OS data were available, and 31 patients were divided into high‐purity and low‐purity groups with the median tumor purity utilized as the cut‐off value. The median survival days in high‐purity and low‐purity group was 1826 and 1713, respectively. Due to small sample size, the Kaplan‐Meier survival analyses in FUSCC cohort did not reveal statistically significant, but the results also indicated that low purity was associated with unfavorable prognosis.

**Table 2:** Univariate and multivariate Cox regression analyses for OS in TCGA cohort

Factors	Univariate analysis		Multivariate analysis	
	HR （95% CI）	*p* value	HR （95% CI）	*p* value
Age	1.9 (1.2‐3)	0.0065		
<56			1	Ref
≥56			2.304 (1.442‐3.682)	<0.001
Sex	1.2 (0.82‐1.6)	0.41		
TNMstage	1.6 (1.3‐2)	<0.001		
1			1	Ref
2			1.498 (0.757‐2.964)	0.246
3			2.483 (1.312‐4.698)	0.005
4			5.228 (2.536‐10.779)	<0.001
Tumor purity	1.5 (1.0‐2.0)	0.026		
High purity			1	Ref
Low purity			1.587 (1.135‐2.220)	0.007

### 3.2 Associations between tumor purity and genomic alterations

**Figure 2:**
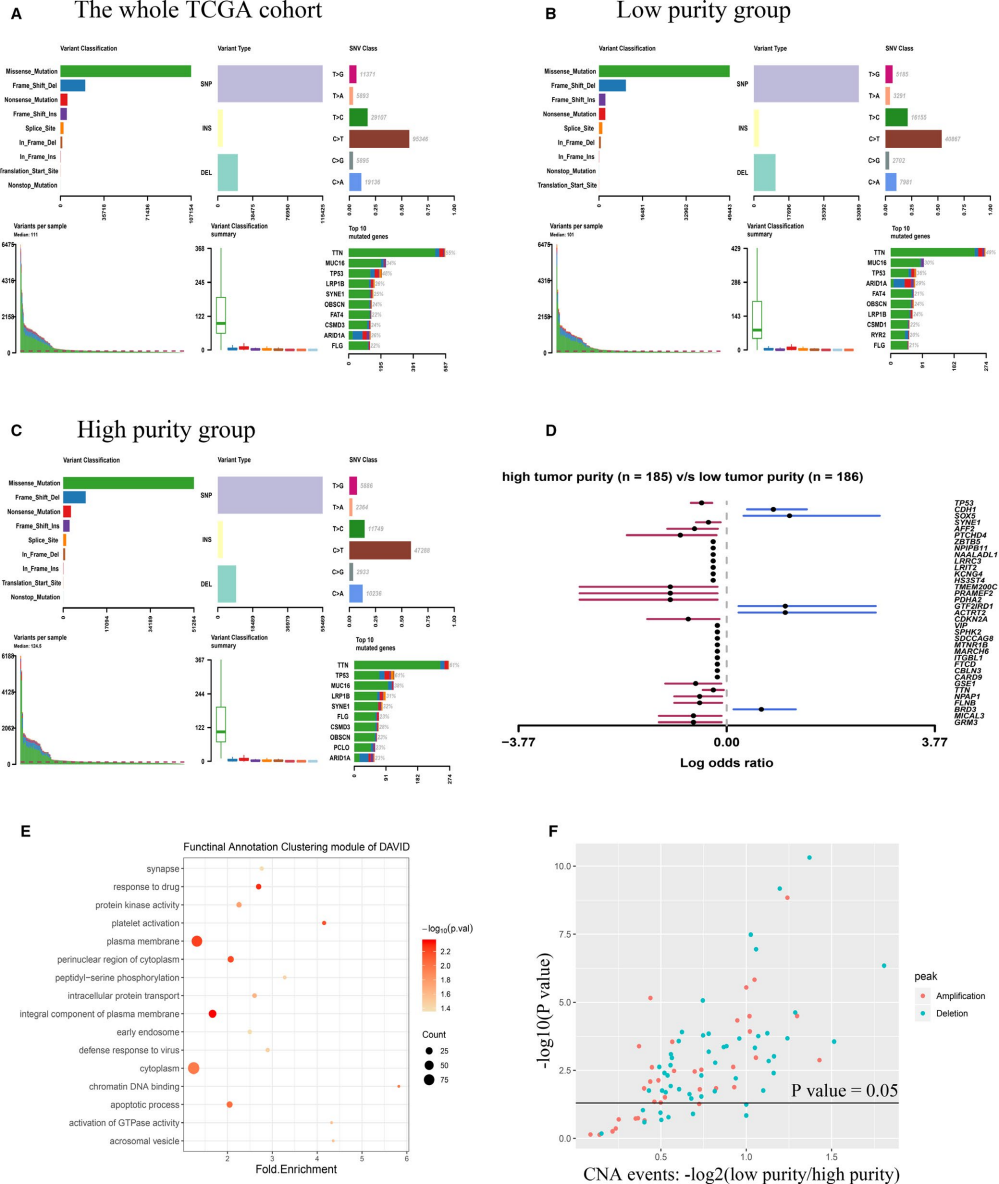
(A) The whole mutation profile in TCGA cohort. (B) Mutation profile in low‐purity groups in TCGA cohort. (C) Mutation profile in high purity groups in TCGA cohort. (D) Differentially mutated genes between low and high‐purity groups in TCGA cohort. (E) The most statistically significant annotation enrichments in genes which were detected more mutations in low‐purity group classified by Functional Annotation Clustering module of DAVID. (F) High‐purity group had more CNA events among all 88 chromosomal locations than low‐purity group

About 371 patients in TCGA cohort with available somatic mutation data were divided into high‐purity and low‐purity groups with the median tumor purity utilized as the cut‐off value. Figure 2A illustrated the summary of the whole mutation profile of TCGA cohort. The median mutation load (number of mutations) in high‐purity and low‐purity groups was 124.5 and 101.0, respectively (Figure 2B,C). However, this difference had not statistical significance (*p* = 0.149).

The most frequently mutated genes among low and high‐purity groups were also shown in Figure 2B,C. Most genes including TP53, SYNE1, AFF2, PTCHD4, and TMEM200C were found significantly more mutated in high‐purity group (all *p* < 0.01), while only five genes were found significantly more mutated in low‐purity group (all *p* < 0.01) (Figure 2D). Moreover, genes which were detected more mutations in low‐purity group were functionally annotated by DAVID, and the significant annotation enrichments were shown in Figure 2E.

Then, we explored the association between tumor purity and CNA events. More CNAs were detected in high‐purity group (low‐purity group vs high‐purity group, 3928 vs 6322 CNAs). Figure 2F showed high‐purity group had more CNA events among all 88 chromosomal locations recognized by GISTIC 2.0 than low‐purity group, and most of them were statistically significant (*p* < 0.05).

### 3.3 Low purity was associated with upregulated epithelial‐mesenchymal transition (EMT) and stemness pathways

**Figure 3:**
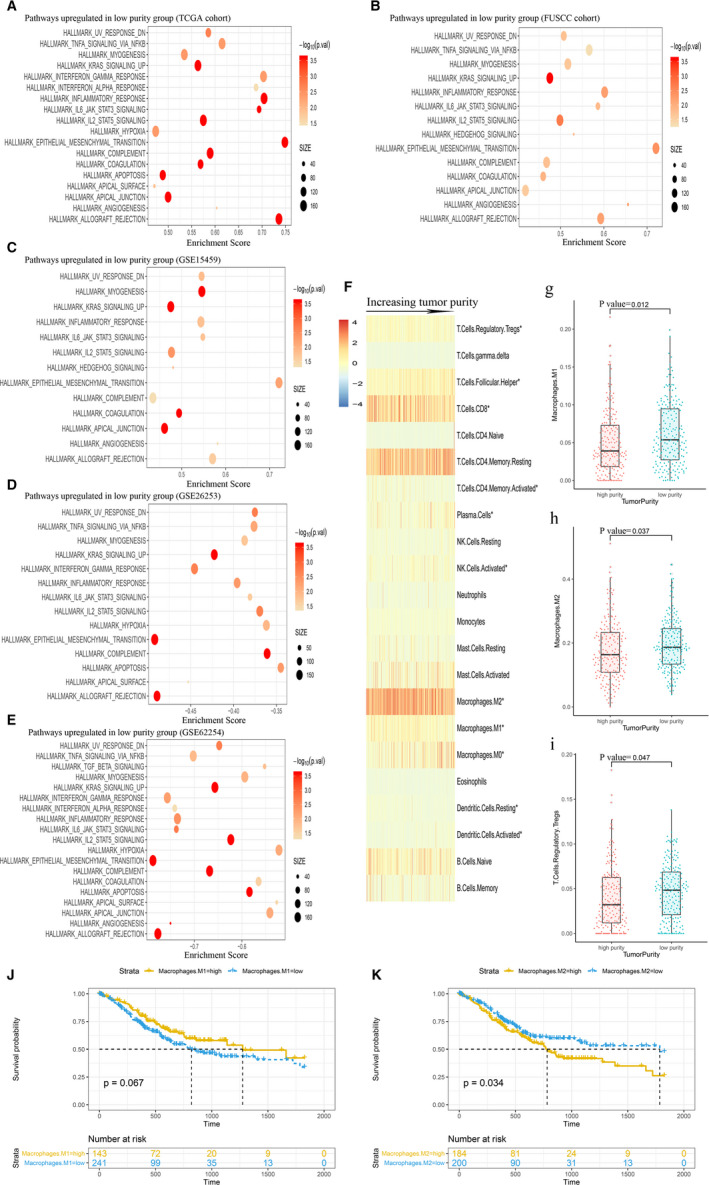
Immune‐related pathways were highly enriched in low‐purity group: (A) TCGA cohort, (B) FUSCC cohort, (C) GSE15459, (D) GSE26253, (e) GSE62254. (F) The distribution of relative proportion of immune cells sorted by increasing purity in TCGA data set. (G, H, and I) The differences between different purity groups in M1 macrophages, M2 macrophages and Tregs infiltrating. Kaplan‐Meier analysis of overall survival showed that more M2 macrophages (J) infiltrating conferred worse prognosis in TCGA cohort, however, more M1 macrophages (k) infiltrating was not significantly associated with OS. * The differences between different purity groups in infiltrating immune cells were statistically significant

We used gene set enrichment analysis (GSEA) to verify the association between tumor purity and biological phenotype. Inflammatory response pathway was detected upregulated in TCGA, FUSCC, GSE15459, GSE26253, and GSE62254 low‐purity group, which indicted that low‐purity group suffered a strengthened immune phenotype. KRAS signaling and EMT pathways which were considered to be able to promote tumor growth and metastasis were found upregulated in all low‐purity groups. Moreover, IL2 ‐ STAT5 signaling and IL6‐ JAK‐STAT3 signaling pathways which were considered as tumor immunosuppressive and stemness‐related pathways were also shown upregulated in TCGA, FUSCC, GSE15459, GSE26253, and GSE62254 low‐purity group (Figure 3A‐E).

### 3.4 Low‐purity group had a higher expression level of immune checkpoints and chemokines

As shown in Table 3, the immune checkpoints, including PD‐L1, PD‐1, LAG‐3, TIGIT, CTLA‐4, and TIM‐3, were all at a higher level in TCGA low‐purity group than the high ones (*p* < 0.05). For patients from FUSCC, the immune checkpoints mentioned above were also at a higher level in low‐purity group, but only the differences in PD‐1, TIGIT, CTLA‐4, and TIM‐3 had statistical significances (*p* < 0.05).

**Table 3:** The expression levels of immune checkpoints and chemokines between different tumor purity groups in TCGA and FUSCC cohorts

	Variable	TCGA‐STAD			FUSCC		
		low purity	high‐purity		low purity	high purity	
		avg.	avg.	*p* value	avg.	avg.	*p* value
Immune checkpoints	PD‐L1	155.72	45.36	0.009	2.29	2.06	0.769
	PD‐1	119.82	42.55	<0.001	2.47	0.65	0.011
	LAG3	312.98	110.78	<0.001	3.59	1.53	0.155
	TIGIT	143.33	44.29	<0.001	3.76	1.53	0.039
	CTLA‐4	4071.11	1114.46	<0.001	6.82	2.59	0.033
	TIM‐3	478.43	157.25	<0.001	7.29	3.47	0.001
Chemokines	CCL1	1.35	0.5	0.001	0.13	0.14	0.927
	CCL2	1160.36	426.03	<0.001	18.84	7.85	0.001
	CCL3	175.93	63.11	<0.001	4.78	0.89	0.136
	CCL5	1850.66	561.2	<0.001	23.45	7.85	0.118
	CCL22	280.43	101.46	<0.001	6.9	2.05	0.018

We also paid attention to the expression level of some chemokines in GC. The Student's *t* test revealed that low‐purity group in TCGA cohort had a higher expression level of chemokines including CCL1, CCL2, CCL3, CCL5, and CCL22 than the high‐purity ones (*p* < 0.05). We also found similar results in FUSCC cohort, although only the differences in CCL2 and CCL22 were statistically significant (Table 3, *p* < 0.05).

### 3.5 Association between tumor purity and tumor‐infiltrating immune cells

We further analyzed the differences in tumor infiltrating immune cells between different tumor purity groups in TCGA cohort. CIBERSORT algorithm was performed to estimate the absolute score and relative proportion of 22 immune cells for each sample. Student's *t* test was performed to find the association between tumor purity and the absolute score of 22 immune cells, and the results showed low‐purity group had more proportion of all 22 immune cells including CD8 T cells, regulatory T cells (Tregs), M1 and M2 macrophages (Additional File 2: Table S1). Heatmap was performed to illustrate the association between the relative proportion of tumor infiltrating immune cells and the increasing tumor purity (Figure 3F). The low‐purity group had more proportion of M1 macrophages, M2 macrophages, and regulatory T cells (Tregs) infiltrating (Figure 3G‐I, *p* < 0.05). We also validated our findings using xCell algorithm (Additional File 3: Table S2), and the results also showed that low‐purity group had more proportion of M1 macrophages (*p* < 0.05), M2 macrophages (*p* < 0.05), and Tregs (*p* = 0.164) infiltrating. Since M1 macrophages and M2 macrophages play different roles in tumor immune response, we further performed survival analyses to find if the two different infiltrating macrophages contributed to clinical outcome in patients with GC. The results showed that the proportion of M1 macrophages was not significantly associated with OS (Figure 3J). However, the proportion of M2 macrophages was shown as an indicator for poor prognosis (*p* < 0.05, Figure 3K).

### 3.6 Association between tumor purity and immune subtypes

**Figure 4:**
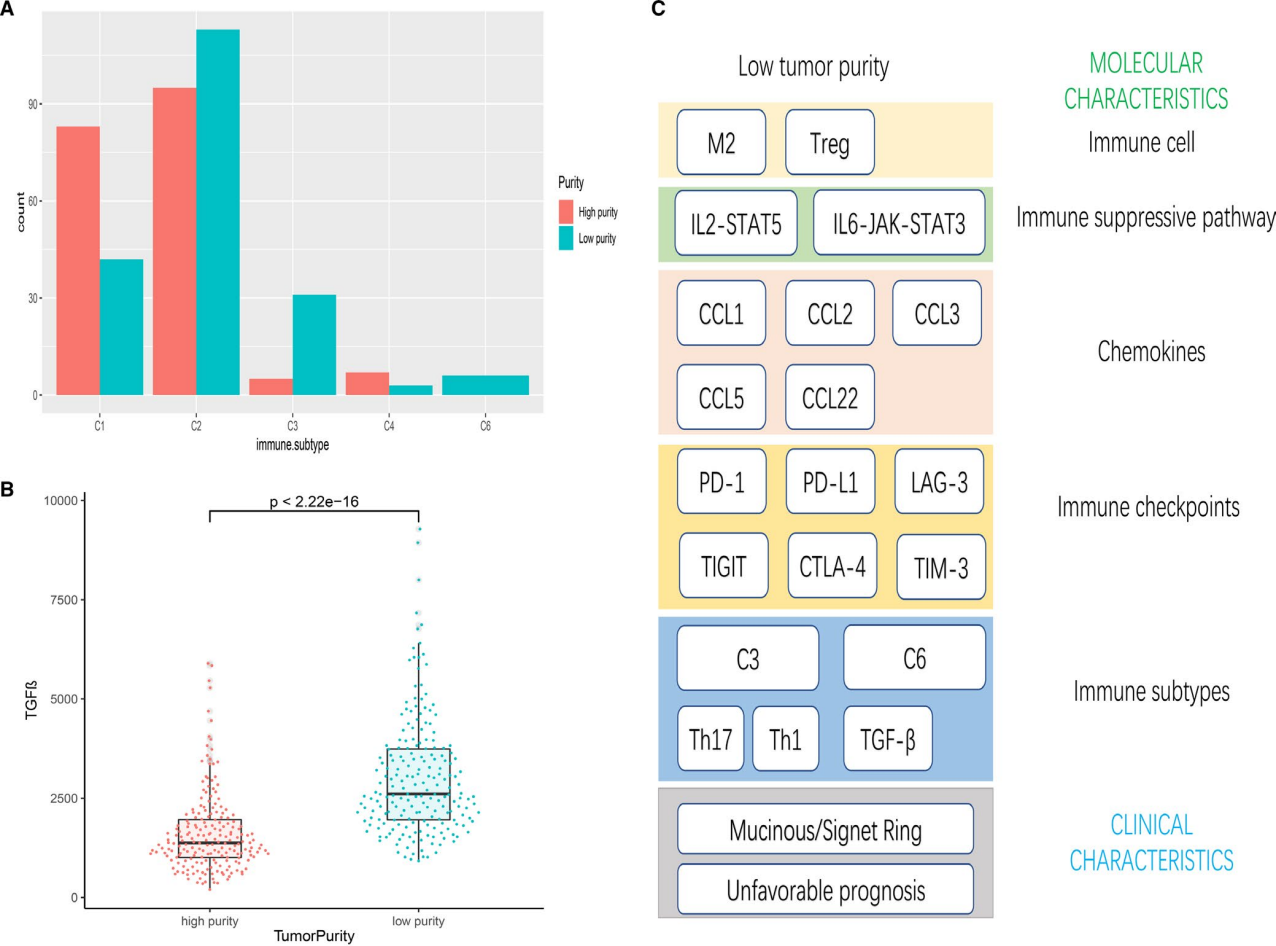
(A) Tumor purity characteristics of immune subtypes in TCGA cohort. (B) The expression of TGF‐β between different purity groups. (C) Summary of characteristics of low tumor purity

A recent published study analyzed tumor samples in TCGA data set and proposed subdividing tumors into six immune subtypes.^7^ For the purpose to explore the underlaying mechanism for tumor purity affecting OS, we performed chi‐square test to find the differences in classification of subtypes according to different tumor purity. The results were showed in Figure 4a. Patients with low‐purity purity were more likely to be classified as C3 (elevated Th17 and Th1 genes, low to moderate tumor cell proliferation, and lower levels of aneuploidy and overall somatic copy number alterations) and C6 (highest TGF‐β signature and a high lymphocytic infiltrate with an even distribution of type I and type II T cells) subtypes (*p* < 0.05). Notably, all patients belonged to C6 subtype had a low‐purity, and C6 subtype had the least favorable outcome according to the published study. Furthermore, we found low‐purity group had a higher expression of TGF‐β (*p* < 0.05, Figure 4b).

## 4 DISCUSSION

In the early studies,^8^ pathologists speculated tumor purity through visual evaluation, and the results was highly depended on the experience of pathologists. With the development of genomics, several computational methods to determine tumor purity were introduced to us, which made the measurement of tumor purity more objective and accurate. According to a comparative study^9^ which compared ESTIMATE, ABSOLUTE, lekocytes unmethylation for purity (LUMP) and immunohistochemistry (IHC), a high concordance was shown between these methods. Therefore, we selected ESTIMATE algorithm for our study because its compatibility in RNA‐Seq and microarray files. The high tumor purity (98.58%‐100.00%) in the 37 GC cell lines revealed that the ESTIMATE algorithm has a perfect robustness in calculating tumor purity in GC. We revealed that tumor purity was strongly associated with clinical and genomic characteristics. Low purity was an independent unfavorable prognostic indicator of OS in GC regardless of age and TNM stage, which consistent with the previous studies in other tumors.^4^, ^5^, ^10^

Genes with higher mutation rate in low‐purity group were functionally annotated by DAVID. The most statistically significant annotation enrichments including protein kinase activity and activation of GTPase activity which may promote tumor growth and metastasis and partially explain the unfavorable prognosis in low‐purity group.^11^, ^12^

Our study also showed high‐purity group had more CNA events among all chromosomal locations. According to a previous study,^13^ power to detect CNAs is highly dependent on the tumor purity, because that large fraction of copy‐neutral DNA from noncancerous cells in low‐purity tumors will significantly decrease the signal/noise ratio of CNAs. The results of our study have validated this point. Therefore, tumor purity is a significant factor that should be considered when we evaluate the CNAs of a patient in the clinical situation.

The high absolute score of tumor‐infiltrating immune cells in the low‐purity group revealed that low‐purity tumors recruited more all kinds of tumor‐infiltrating immune cells including immune promoting and suppressing cells and the relative proportion showed the final resultant force of increased tumor‐infiltrating immune cells. Previous studies have showed Tregs suppress antitumor immune response by impairing cell‐mediated immune responses to tumors and further promote disease progression.^14^ In general, M2 macrophages secrete immune suppressive cytokines and chemokines and develop the protumoral effect.^15^, ^16^ Interestingly, several studies^17^, ^18^, ^19^, ^20^ revealed that M2 tumor‐associated macrophages may trigger a rise of the intratumoral Treg population and lead to poor prognosis. Furthermore, the higher absolute score of CD8 T cells in low tumor purity indicates that anti‐PD‐1/PD‐L1 therapy may benefit low‐purity patients. However, the existence of higher Tregs and M2 macrophages may weaken the effect of immunotherapy. Therefore, anti‐Tregs and anti‐M2 macrophages combine with anti‐PD‐1/PD‐L1 therapy may be a better choice for low‐purity group patients. Survival analyses showed the proportion of M2 macrophages presented negative prognostic value, which may partially explain the unfavorable prognosis in low‐purity group. M1 macrophages were considered to have the pro‐inflammatory and antitumoral effects.^21^ However, survival analyses showed the relative proportion of M1 macrophages was not significantly associated with OS, which indicated the high proportion M1 macrophages in low‐purity group was insufficient to change the prognosis.

GSEA results revealed immune‐related pathways were highly enriched in low‐purity group. Moreover, EMT pathways were found upregulated in low‐purity group and the result may explain the unfavorable prognosis of low tumor purity group and revealed that low‐purity tumors were more likely to metastasize. More importantly, IL2‐STAT5 signaling and IL6‐JAK‐STAT3 signaling gene sets were found upregulated in low‐purity group. A recent study pointed that IL2 and downstream transcription factor STAT5 are important for maintaining immunosuppressive Tregs homeostasis and function.^22^ This result is consistent with the high proportion of Tregs in low‐purity group, and may further explain the reasons of the unfavorable prognosis in low‐purity group. As for the IL6‐JAK‐STAT3 signaling, studies showed it involved in tumor growth, metastasis, and the immune escaping.^23^, ^24^, ^25^, ^26^, ^27^ Therefore, it may be a reason for unfavorable prognosis in low‐purity group. Both IL2‐STAT5 signaling and IL6‐JAK‐STAT3 signaling may become potential immunotherapeutic targets for low‐purity gastric tumors.

Low‐purity group had a higher expression level of chemokines. Many studies pointed that CCL2 is the major determinant of macrophage content in tumors.^28^, ^29^ Besides, CCL3 and CCL5 also take part in recruiting M2 macrophages to tumors.^29^, ^30^ Furthermore, previous studies^31^, ^32^, ^33^ indicated that CCL1, CCL2, CCL5, and CCL22 play an important role in recruiting Tregs to tumors. These results were consistent with above analysis of tumor‐infiltrating immune cells. Thus, these findings may indicate that the high expression level of chemokines mentioned above recruited immune suppressive cells and help tumor immune escape and resulted in the unfavorable prognosis in low‐purity group. The results indicated that immunotherapy against these chemokines may bring a better clinical outcome for low tumor purity GC patients. Remarkably, the immune checkpoint gene were at a higher expression level in the low‐purity group than those in the high‐purity group. As is known to all, signaling through immune checkpoint receptors may lead to T cell exhaustion and function as immune escape mechanisms in cancer.^34^, ^35^ Therefore, the immunotherapy drugs add to the traditional chemotherapy may become a new choice to improve the prognosis for the low tumor purity patients. Further validation for our findings is needed.

Thorsson et al. divided samples in TCGA into six immune subtypes (C1‐C6).^7^ Here, we found that low tumor purity group was significantly associated with C3 and C6 immune subtypes. The previous study^7^ also indicated that an increased value of macrophage regulation or TGF‐β led to worse outcome in C3. Since the low‐purity group in GC was associated with more M2 macrophages infiltrating and more TGF‐β expression, it may partly explain the unfavorable prognosis in low‐purity group. Notably, all patients belonged to C6 subtype had a low‐purity. The feature of C6 subtype is the highest TGF‐β signature and a high lymphocytic infiltrate with an even distribution of type I and type II T cells. Our study also found that low‐purity group in GC had a high expression level of TGF‐β. Several studies^36^, ^37^ illustrated that TGF‐β was correlated with migration, invasion, and distant metastasis of gastric cancer cells, which might partially explain the unfavorable prognosis of low tumor purity group. Moreover, weakening the function of TGF‐β can help inhibit the metastasis of GC.^36^ Perhaps the immunotherapy targets TGF‐β will improve the prognosis of low‐purity GC patients.

As shown in Figure 4C, we summarized the characteristics of low‐purity GC. Low tumor purity in GC was associated with more M2 macrophages and Tregs infiltrating, upregulated tumor immunosuppressive pathway and a higher expression level of immune checkpoints and chemokines, which were all contribute to cancer immune escape. These results may indicate that immune escape is an underlying mechanism for unfavorable prognosis in low tumor purity group, and perhaps low purity GC patients will benefit more from immunotherapy.

## 5 CONCLUSIONS

In a word, our study revealed that tumor purity plays an important role in prediction of prognosis and genomic conditions in GC. Low purity in GC was associated with enhanced immune evasion and poor prognosis, which indicated that low‐purity GC patients may benefit more from immunotherapy. Further investigations need to be performed on tumor purity in order to get a better comprehension in TME and make a better clinical decision.

## ETHICS APPROVAL AND CONSENT TO PARTICIPATE

Written informed consent was obtained from all participants for their tissues to be utilized for this work, and the application of the patient tissue sample and the study has been approved by the FUSCC ethics committee.

## CONFLICT OF INTEREST

The authors declare that they have no competing interests.

## AUTHOR CONTRIBUTIONS

All authors were involved in the conception of the study, and revised and approved the final manuscript. All authors take the responsibility for publishing this paper. Analysis, Computation, and Software: J.Z. and Z.G. RNA Sequencing of patients in FUSCC cohort: J.Z. Supervision: W.G. Writing: J.Z. and Z.G. Editing the manuscript: Z.G.

## Supporting information

Table S1

Table S2

Document S1

## Data Availability

The data sets generated and analyzed during the current study are available in the TCGA, GEO, and CCLE repository, TCGA: https://portal.gdc.cancer.gov GEO: https://www.ncbi.nlm.nih.gov/geo CCLE: https://portals.broadinstitute.org/ccle. FUSCC cohort data used and analyzed during the current study are available from the corresponding author on reasonable request.
