# Supplementary material for: Tumor purity as a prognosis and immunotherapy relevant feature in gastric cancer
Source: Cancer Med. 2020 Oct 8;9(23):9052–63. doi: 10.1002/cam4.3505 (PMC7724479; doi:10.1002/cam4.3505)
Supplement: Supplementary file 3 — Document S1 [file CAM4-9-9052-s003.docx]

**Supporting Information Document S1**

**Materials and methods**

**Study samples**

407, 200, 432, 300 and 34 gastric cancer patients form TCGA dataset, GSE15459, GSE26253, GSE62254 and FUSCC respectively with available data of RNA sequencing and 34 gastric cancer patients from FUSCC were enrolled in our study. Our outcome of interest is overall survival (OS) and the end points were the follow-up time up to 5 years or the death of the patient. Patient characteristics of the two cohorts were described in Table 1. Furthermore, we obtained RNA-Seq data of 37 GC cell lines from Cancer Cell Line Encyclopedia (CCLE, https://portals.broadinstitute.org/ccle) in order to validate the ESTIMATE algorithm. Written informed consent was obtained from all participants for their tissues to be utilized for this work, and the application of the patient tissue sample and the study has been approved by the FUSCC ethics committee.

**RNA Sequencing**

To get the RNA-seq data in FUSCC cohort, we treated the total RNA samples (1 μg) of GC tissue with Ribo-off rRNA Depletion Kit (Vazyme) before constructing the RNA-seq libraries. VAHTS Total RNA-seq (H/M/R) Library Prep Kit for Illumina (Vazyme) were utilized to prepare RNA-seq libraries following the manufacturer’s instructions. Furthermore, ribosome depleted RNA samples (approximately 100 ng) were fragmented and then used for first- and second-strand cDNA synthesis with random hexamer primers. The ends of the cDNA fragments were repaired by DNA End Repair Kit. Then the cDNA fragments were modified with Klenow to add an A at the 3’ end of the DNA fragments, and finally ligated to adapters. We subjected purified dsDNA to 12 cycles of PCR amplification, and sequenced the libraries by Illumina sequencing platform on a 150 bp paired-end run. Sequencing reads from RNA-seq data were aligned using the spliced read aligner HISAT2, which was supplied with the Ensembl human genome assembly (Genome Reference Consortium GRCh38) as the reference genome. Gene expression levels were calculated by the FPKM (fragments per kilobase of transcript per million mapped reads). We utilized the GENCODE (v25) database to get annotations of mRNA in the human genome.

**Bioinformatic analysis**

We downloaded 407 gastric patients’ TCGA level 3 RNA-Seq version 2 RSEM data from the cBioPortal for Cancer Genomics (http://www.cbioportal.org/). Among the 407 patients, 371 patients’ somatic mutation data was available, and we also downloaded the somatic mutation data from the cBioPortal for Cancer Genomics for genomic analysis. 200, 432 and 300gastric cancer patients’ RNA-Seq data form GSE15459, GSE26253 and GSE62254 respectively were downloaded from Gene Expression Omnibus (GEO, https://www.ncbi.nlm.nih.gov/geo/). 37 GC cell lines’ RNA-Seq data were downloaded from Cancer Cell Line Encyclopedia. The RNA-Seq data of 34 gastric cancer patients from FUSCC were collected for validation. The gene expression in TCGA, GEO and FUSCC cohorts was estimated using the RNA-Seq by the Expectation Maximization (RESM) and fragments per kilobase of transcript per millon mapped reads) (FPKM) method. Therefore, the impact of different gene lengths and sequencing discrepancies from the expression calculation will be removed and we could use the calculated gene expression data to compare the differences in gene expression amidst samples.

ESTIMATE [7] was a widely used algorithm for tumor purity prediction. We used ESTIMATE R package to calculate stromal and immune scores, which formed the basis for the ESTIMATE score to infer tumor purity in gastric tumor tissue. CIBERSORT algorithm was utilized to estimate the absolute score and relative proportion of 22 immune cells for each sample in TCGA cohort. We utilized GISTIC 2.0 to analyze the copy number alterations (CNA) events. The results of cell type enrichment analysis for TCGA data using xCell were downloaded from https://xcell.ucsf.edu/. DAVID’s Functional Annotation Clustering module under the conditions of Homo sapiens of species was used to classify gene list into functional related gene groups. Gene set enrichment analysis (GSEA) was performed by the GSEA software v.3.0 utilizing Molecular Signatures Database (MSigDB) v6.2 with 1000 permutations.

**Statistical analysis**

All of our analyses were conducted using R software version 3.5.2 (https://www.r-project.org/) and SPSS 20.0 (SPSS Inc., Chicago, IL, USA). Kaplan-Meier analyses was used to evaluate the relationship between different purity groups and overall survival (OS). Univariate and multivariate Cox regression analyses were performed to identify independent prognostic factors. Student’s t-tests was used to compare variables between groups. Correlations between categorical variables were evaluated by chi-square analyses. Statistically significant was admitted if a two-sided P value <0.05.
